# Supplementary material for: A single mutation in Taiwanese H6N1 influenza hemagglutinin switches binding to human‐type receptors
Source: EMBO Mol Med. 2017 Jul 10;9(9):1314–25. doi: 10.15252/emmm.201707726 (PMC5582370; doi:10.15252/emmm.201707726)
Supplement: Supplementary file 1 — Appendix [file EMMM-9-1314-s001.pdf]

APPENDIX
SUPPORTING INFORMATION

A single mutation in Taiwanese H6N1 influenza hemagglutinin switches binding to human-type receptors

Robert P de Vries[1,2#], Netanel Tzarum[3#], Wenjie Peng[1#], Andrew Thompson[1] Iresha N Ambepitiya Wickramasinghe[4], Alba T. Torrents de la Pena[5], Marielle J. van Breemen[5], Kim M Bouwman[4], Xueyong Zhu[3], Ryan McBride[1], Wenli Yu[3], Rogier W Sanders[5,6], Monique H Verheije[4], Ian A Wilson[3,7*] and James C Paulson[1*].

[1] Departments of Molecular Medicine & Immunology and Microbiology, The Scripps Research Institute, 10550 North Torrey Pines Road, La Jolla, CA 92037, USA

[2] Department of Chemical Biology and Drug Discovery, Utrecht Institute for Pharmaceutical Sciences, Utrecht University, 3584 CG Utrecht, The Netherlands

[3] Department of Integrative Structural and Computational Biology, The Scripps Research Institute, The Scripps Research Institute, 10550 North Torrey Pines Road, La Jolla, CA 92037, USA

[4] Pathology Division, Department of Pathobiology, Faculty of Veterinary Medicine, Utrecht University, 3584 CL Utrecht, The Netherlands

[5] Department of Medical Microbiology, Academic Medical Center, University of Amsterdam, 1105 AZ Amsterdam, The Netherlands

[6] Department of Microbiology and Immunology, Weil Medical College of Cornell University, New York, NY 10065, USA

[7] Skaggs Institute for Chemical Biology, The Scripps Research Institute, 10550 North Torrey Pines Road, La Jolla, CA 92037, USA

[#] These authors contributed equally

* To whom correspondence should be addressed. E-mail: wilson@scripps.edu, jpaulson@scripps.edu

Table of contents

Table S1. Glycans imprinted on the sialoside array

Table S2. Data Collection and Refinement Statistics for H6 HA G225D and Receptor Analog Complexes

Table S3. Top hits of A/Taiwan/2/13 and A/Taiwan/2/13 G225D with the structures shown.

Table S4. Apparent avidities of the A/Taiwan/2/13 and the G225D mutant.

Figure S1. Receptor binding specificity of H6N1 wild-type and G225D mutant HAs expressed in insect cells.

Figure S2. Analysis of plant lectin binding to chicken and human trachea epithelial cells.

Figure S3. Electron density for receptor analogs in the H6 G225D HA crystal structures

Figure S4. Structural comparison of human-type receptor conformations.

Figure S5. Crystal structures of the H6 G225D mutant in complex with avian receptor analogues.

References

Table S1. Glycans imprinted on the sialoside array. Non-sialylated controls #1-10 in gray, avian-type receptors #11-79 in white and human-type receptors #80-135 in black. The Common Name column lists glycan sequences and linkers in IUPAC format except for abbreviations of synthetic structures 68, 132-135 described elsewhere. The Structure column represents the glycan portion of the structure in symbol format (Note: linkers are not consistently annotated, see Common Name for linkers). Purple diamonds represent sialic acid, yellow circles represent galactose, blue circles represent glucose, green circles represent mannose, yellow squares represent N-acetyl-galactosamine, blue squares represent N-acetyl-glucosamine, and red triangles represent fucose.

Glycan #	Common Name	Structure
1	Galβ(1-4)GlcNAcβ-ethyl-NH2	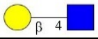
2	Galβ(1-4)GlcNAcβ(1-3)Galβ(1-3)GalNAcα-Thr-NH2	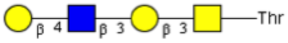
3	Galβ(1-4)GlcNAcβ(1-6)[Galβ(1-3)]-GalNAcα-Thr-NH2	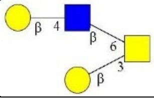
4	Galβ(1-4)GlcNAcβ(1-3)GalNAcα-Thr-NH2	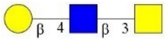
5	Galβ(1-4)GlcNAcβ(1-3)[Galβ(1-4)GlcNAcβ(1-6)]-GalNAcα-Thr-NH2	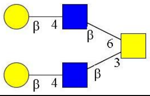
6	Galβ(1-4)GlcNAcβ(1-6)GalNAcα-Thr-NH2	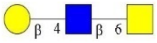
7	Galβ(1-4)GlcNAcβ(1-2)Manα (1-3)[Galβ(1-4)GlcNAcβ(1-2)Manα(1-6)]-Manβ(1-4)GlcNAcβ(1-4)GlcNAcβ-Asn-NH2	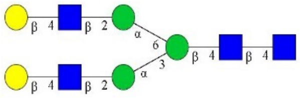
8	Galβ(1-4)GlcNAcβ(1-2)Manα(1-3)[Galβ(1-4)GlcNAcβ(1-2)Manα(1-6)]-Manβ(1-4)GlcNAcβ(1-4)[Fucα(1-6)]-GlcNAcβ-Asn-Ser-Thr-NH2	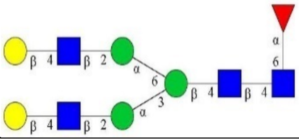
9	Galβ(1-4)GlcNAcβ(1-2)Manα(1-3){Galβ(1-4)GlcNAcβ(1-2)[Galβ(1-4)GlcNAcβ(1-2)]-Manα(1-6)}-Manβ(1-4)GlcNAcβ(1-4)GlcNAcβ-Asn-Lys-NH2	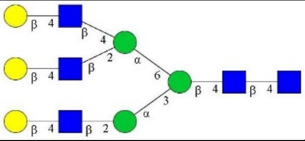
10	Galβ(1-4)GlcNAcβ(1-2)Manα(1-3){Galβ(1-4)GlcNAcβ(1-2)[Galβ(1-4)GlcNAcβ(1-2)]-Manα(1-6)}-Manβ(1-4)GlcNAcβ(1-4)[Fucα(1-6)]-GlcNAcβ-(Lys-Val-Ala)Asn-Lys-Thr-NH2	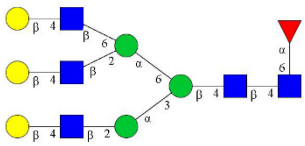
11	NeuAcα(2-3)Galβ(1-4)6-O-sulfo-GlcNAcβ-propyl-NH2	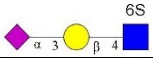
12	NeuAcα(2-3)Galβ(1-4)[Fucα(1-3)]-6-O-sulfo-GlcNAcβ-propyl-NH2	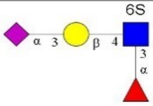
13	NeuAcα(2-3)6-O-sulfo-Galβ(1-4)GlcNAcβ-ethyl-NH2	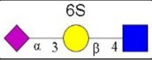
14	NeuAcα(2-3)6-O-sulfo-Galβ(1-4)[Fucα(1-3)]-GlcNAcβ-propyl-NH2	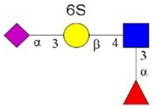
15	NeuAcα(2-3)Galβ(1-3)6-O-sulfo-GlcNAcβ-propyl-NH2	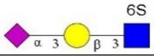

Glycan #	Common Name	Structure
16	NeuAcα(2-3)Galβ(1-4)Glcβ-ethyl-NH2	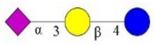
17	NeuAcα(2-3)Galβ(1-4)GlcNAcβ-ethyl-NH2	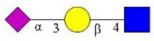
18	NeuAcα(2-3)Galβ(1-4)GlcNAcβ(1-3)Galβ(1-4)GlcNAcβ-ethyl-NH2	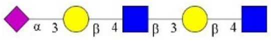
19	NeuAcα(2-3)Galβ(1-4)GlcNAcβ(1-3)Galβ(1-4)GlcNAcβ(1-3)Galβ(1-4)GlcNAcβ-ethyl-NH2	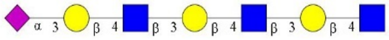
20	NeuAcα(2-3)GalNAcβ(1-4)GlcNAcβ-ethyl-NH2	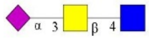
21	NeuAcα(2-3)Galβ(1-3)GlcNAcβ-ethyl-NH2	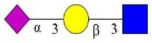
22	NeuAcα(2-3)Galβ(1-3)GlcNAcβ(1-3)Galβ(1-4)GlcNAcβ-ethyl-NH2	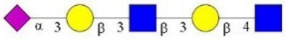
23	NeuAcα(2-3)Galβ(1-3)GlcNAcβ(1-3)Galβ(1-3)GlcNAcβ-ethyl-NH2	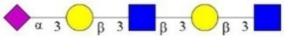
24	NeuAcα(2-3)Galβ(1-3)GalNAcβ(1-3)Galα(1-4)Galβ(1-4)Glcβ-ethyl-NH2	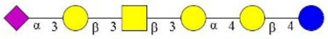
25	NeuAcα(2-3)Galβ(1-3)GalNAcα-Thr-NH2	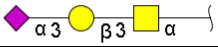
26	NeuAcα(2-3)Galβ(1-4)GlcNAcβ(1-3)Galβ(1-3)GalNAcα-Thr-NH2	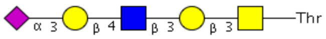
27	NeuAcα(2-3)Galβ(1-4)GlcNAcβ(1-3)Galβ(1-4)GlcNAcβ(1-3)Galβ(1-3)GalNAcα-Thr-NH2	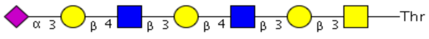
28	NeuAcα(2-3)Galβ(1-4)GlcNAcβ(1-3)Galβ(1-4)GlcNAcβ(1-3)Galβ(1-3)GalNAcα-Thr-NH2	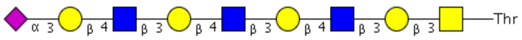
29	NeuAcα(2-3)Galβ(1-4)GlcNAcβ(1-3)Galβ(1-4)GlcNAcβ(1-3)Galβ(1-4)GlcNAcβ(1-3)Galβ(1-3)GalNAcα-Thr-NH2	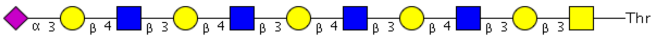
30	NeuAcα(2-3)Galβ(1-4)GlcNAcβ(1-3)Galβ(1-4)GlcNAcβ(1-3)Galβ(1-4)GlcNAcβ(1-3)Galβ(1-4)GlcNAcβ(1-3)Galβ(1-3)GalNAcα-Thr-NH2	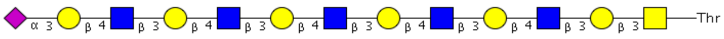
31	NeuAcα(2-3)Galβ(1-3)[GlcNAcβ(1-6)]-GalNAcα-Thr-NH2	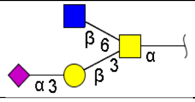
32	NeuAcα(2-3)Galβ(1-4)GlcNAcβ(1-6)[Galβ(1-3)]-GalNAcα-Thr-NH2	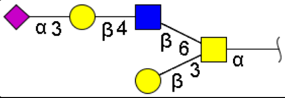
33	NeuAcα(2-3)Galβ(1-4)GlcNAcβ(1-3)Galβ(1-4)GlcNAcβ(1-6)[Galβ(1-3)]-GalNAcα-Thr-NH2	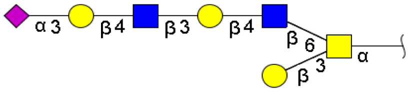
34	NeuAcα(2-3)Galβ(1-4)GlcNAcβ(1-3)Galβ(1-4)GlcNAcβ(1-3)Galβ(1-4)GlcNAcβ(1-6)[Galβ(1-3)]-GalNAcα-Thr-NH2	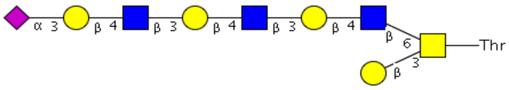
35	NeuAcα(2-3)Galβ(1-4)GlcNAcβ(1-3)Galβ(1-4)GlcNAcβ(1-3)Galβ(1-4)GlcNAcβ(1-3)Galβ(1-4)GlcNAcβ(1-6)[Galβ(1-3)]-GalNAcα-Thr-NH2	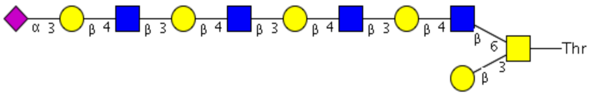

Glycan #	Common Name	Structure
36	NeuAcα(2-3)Galβ(1-4)GlcNAcβ(1-3)Galβ(1-4)GlcNAcβ(1-3)Galβ(1-4)GlcNAcβ(1-3)Galβ(1-4)GlcNAcβ(1-3)Galβ(1-4)GlcNAcβ(1-6)[Galβ(1-3)]-GalNAcα-Thr-NH2	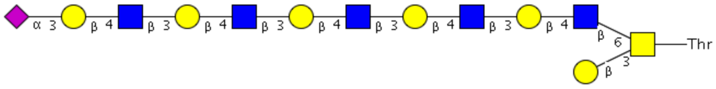
37	NeuAcα(2-3)Galβ(1-4)GlcNAcβ(1-3)Galβ(1-4)GlcNAcβ(1-3)Galβ(1-4)GlcNAcβ(1-3)Galβ(1-4)GlcNAcβ(1-6)[NeuAcα(2-3)Galβ(1-4)GlcNAcβ(1-3)Galβ(1-4)GlcNAcβ(1-3)Galβ(1-4)GlcNAcβ(1-3)Galβ(1-3)]-GalNAcα-Thr-NH2	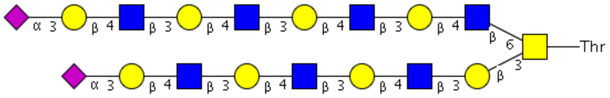
38	NeuAcα(2-3)Galβ(1-4)GlcNAcβ(1-3)Galβ(1-4)GlcNAcβ(1-3)Galβ(1-4)GlcNAcβ(1-3)Galβ(1-4)GlcNAcβ(1-3)Galβ(1-4)GlcNAcβ(1-6)[NeuAcα(2-3)Galβ(1-4)GlcNAcβ(1-3)Galβ(1-4)GlcNAcβ(1-3)Galβ(1-4)GlcNAcβ(1-3)Galβ(1-4)GlcNAcβ(1-3)Galβ(1-3)]-GalNAcα-Thr-NH2	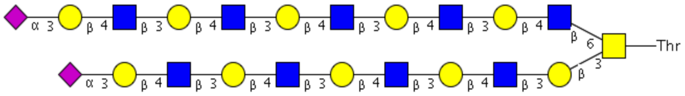
39	NeuAcα(2-3)Galβ(1-4)GlcNAcβ(1-3)GalNAcα-Thr-NH2	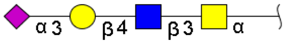
40	NeuAcα(2-3)Galβ(1-4)GlcNAcβ(1-3)Galβ(1-4)GlcNAcβ(1-3)GalNAcα-Thr-NH2	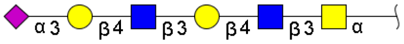
41	NeuAcα(2-3)Galβ(1-4)GlcNAcβ(1-3)Galβ(1-4)GlcNAcβ(1-3)Galβ(1-4)GlcNAcβ(1-3)GalNAcα-Thr-NH2	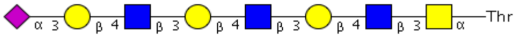
42	NeuAcα(2-3)Galβ(1-4)GlcNAcβ(1-3)Galβ(1-4)GlcNAcβ(1-3)Galβ(1-4)GlcNAcβ(1-3)Galβ(1-4)GlcNAcβ(1-3)GalNAcα-Thr-NH2	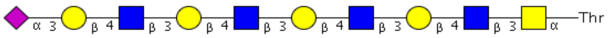
43	NeuAcα(2-3)Galβ(1-4)GlcNAcβ(1-3)Galβ(1-4)GlcNAcβ(1-3)Galβ(1-4)GlcNAcβ(1-3)Galβ(1-4)GlcNAcβ(1-3)Galβ(1-4)GlcNAcβ(1-3)GalNAcα-Thr-NH2	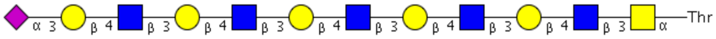
44	NeuAcα(2-3)Galβ(1-4)GlcNAcβ(1-3)[NeuAcα(2-3)Galβ(1-4)GlcNAcβ(1-6)]-GalNAcα-Thr-NH2	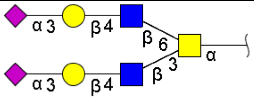
45	NeuAcα(2-3)Galβ(1-4)GlcNAcβ(1-3)Galβ(1-4)GlcNAcβ(1-3)[NeuAcα(2-3)Galβ(1-4)GlcNAcβ(1-3)Galβ(1-4)GlcNAcβ(1-6)]-GalNAcα-Thr-NH2	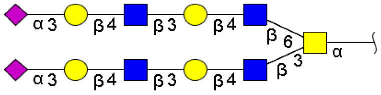
46	NeuAcα(2-3)Galβ(1-4)GlcNAcβ(1-3)Galβ(1-4)GlcNAcβ(1-3)Galβ(1-4)GlcNAcβ(1-3)[NeuAcα(2-3)Galβ(1-4)GlcNAcβ(1-3)Galβ(1-4)GlcNAcβ(1-3)Galβ(1-4)GlcNAcβ(1-6)]-GalNAcα-Thr-NH2	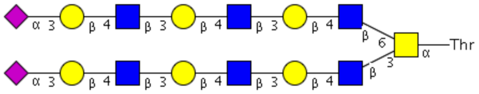
47	NeuAcα(2-3)Galβ(1-4)GlcNAcβ(1-3)Galβ(1-4)GlcNAcβ(1-3)Galβ(1-4)GlcNAcβ(1-3)Galβ(1-4)GlcNAcβ(1-3)[NeuAcα(2-3)Galβ(1-4)GlcNAcβ(1-3)Galβ(1-4)GlcNAcβ(1-3)Galβ(1-4)GlcNAcβ(1-3)Galβ(1-4)GlcNAcβ(1-6)]-GalNAcα-Thr-NH2	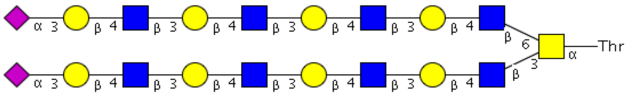

Glycan #	Common Name	Structure
48	NeuAcα(2-3)Galβ(1-4)GlcNAcβ(1-3)Galβ(1-4)GlcNAcβ(1-3)Galβ(1-4)GlcNAcβ(1-3)Galβ(1-4)GlcNAcβ(1-3)Galβ(1-4)GlcNAcβ(1-3)[NeuAcα(2-3)Galβ(1-4)GlcNAcβ(1-3)Galβ(1-4)GlcNAcβ(1-3)Galβ(1-4)GlcNAcβ(1-3)Galβ(1-4)GlcNAcβ(1-3)Galβ(1-4)GlcNAcβ(1-6)]-GalNAcα-Thr-NH2	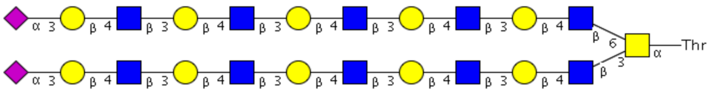
49	NeuAcα(2-3)Galβ(1-4)GlcNAcβ(1-3)Galβ(1-4)GlcNAcβ(1-3)Galβ(1-4)GlcNAcβ(1-3)Galβ(1-4)GlcNAcβ(1-6)GalNAcα-Thr-NH2	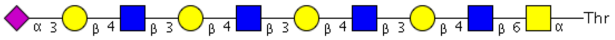
50	NeuAcα(2-3)Galβ(1-4)GlcNAcβ(1-3)Galβ(1-4)GlcNAcβ(1-3)Galβ(1-4)GlcNAcβ(1-3)Galβ(1-4)GlcNAcβ(1-3)Galβ(1-4)GlcNAcβ(1-6)GalNAcα-Thr-NH2	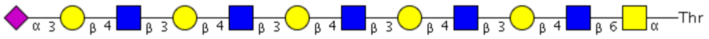
51	NeuAcα(2-3)Galβ(1-3)GlcNAcβ(1-3)Galβ(1-4)GlcNAcβ(1-6)[NeuAcα(2-3)Galβ(1-3)GlcNAcβ(1-3)] Galβ(1-4)GlcNAcβ-ethyl-NH2	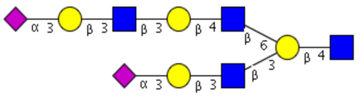
52	NeuAcα(2-3)Galβ(1-4)GlcNAcβ(1-3)Galβ(1-4)GlcNAcβ(1-3)Galβ(1-4)GlcNAcβ(1-6)[NeuAcα(2-3)Galβ(1-4)GlcNAcβ(1-3)Galβ(1-4)GlcNAcβ(1-3)] Galβ(1-4)GlcNAcβ-ethyl-NH2	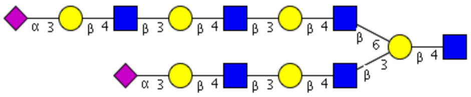
53	NeuAcα(2-3)Galβ(1-4)GlcNAcβ(1-2)Manα(1-3)[NeuAcα(2-3)Galβ(1-4)GlcNAcβ(1-2)Manα(1-6)]-Manβ(1-4)GlcNAcβ(1-4)GlcNAcβ-Asn-NH2	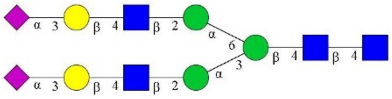
54	NeuAcα(2-3)Galβ(1-4)GlcNAcβ(1-3)Galβ(1-4)GlcNAcβ(1-2)Manα(1-3)[NeuAcα(2-3)Galβ(1-4)GlcNAcβ(1-3)Galβ(1-4)GlcNAcβ(1-2)Manα(1-6)]-Manβ(1-4)GlcNAcβ(1-4)GlcNAcβ-Asn-NH2	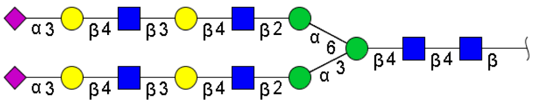
55	NeuAcα(2-3)Galβ(1-4)GlcNAcβ(1-3)Galβ(1-4)GlcNAcβ(1-3)Galβ(1-4)GlcNAcβ(1-2)Manα(1-3)[NeuAcα(2-3)Galβ(1-4)GlcNAcβ(1-3)Galβ(1-4)GlcNAcβ(1-3)Galβ(1-4)GlcNAcβ(1-2)Manα(1-6)]-Manβ(1-4)GlcNAcβ(1-4)GlcNAcβ-Asn-NH2	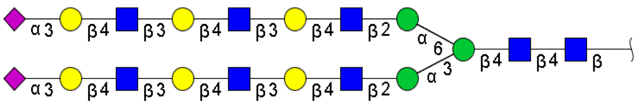
56	NeuAcα(2-3)Galβ(1-4)GlcNAcβ(1-3)Galβ(1-4)GlcNAcβ(1-2)Manα(1-3)[NeuAcα(2-3)Galβ(1-4)GlcNAcβ(1-3)Galβ(1-4)GlcNAcβ(1-2)Manα(1-6)]-Manβ(1-4)GlcNAcβ(1-4)GlcNAcβ-(Lys-Val-Ala)Asn-Lys-Thr-NH2	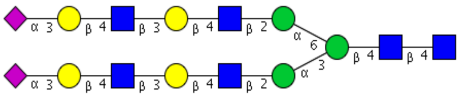
57	NeuAcα(2-3)Galβ(1-4)GlcNAcβ(1-3)Galβ(1-4)GlcNAcβ(1-3)Galβ(1-4)GlcNAcβ(1-2)Manα(1-3)[NeuAcα(2-3)Galβ(1-4)GlcNAcβ(1-3)Galβ(1-4)GlcNAcβ(1-3)Galβ(1-4)GlcNAcβ(1-2)Manα(1-6)]-Manβ(1-4)GlcNAcβ(1-4)GlcNAcβ-(Lys-Val-Ala)Asn-Lys-Thr-NH2	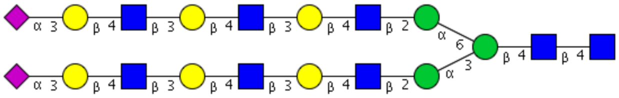

Glycan #	Common Name	Structure
58	NeuAcα(2-3)Galβ(1-4)GlcNAcβ(1-3)Galβ(1-4)GlcNAcβ(1-3)Galβ(1-4)GlcNAcβ(1-3)Galβ(1-4)GlcNAcβ(1-2)Manα(1-3)[NeuAcα(2-3)Galβ(1-4)GlcNAcβ(1-3)Galβ(1-4)GlcNAcβ(1-3)Galβ(1-4)GlcNAcβ(1-3)Galβ(1-4)GlcNAcβ(1-2)Manα(1-6)]-Manβ(1-4)GlcNAcβ(1-4)GlcNAcβ-(Lys-Val-Ala)Asn-Lys-Thr-NH2	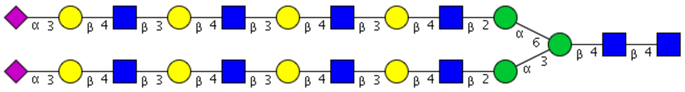
59	NeuAcα(2-3)Galβ(1-4)GlcNAcβ(1-3)Galβ(1-4)GlcNAcβ(1-3)Galβ(1-4)GlcNAcβ(1-3)Galβ(1-4)GlcNAcβ(1-3)Galβ(1-4)GlcNAcβ(1-2)Manα(1-3)[NeuAcα(2-3)Galβ(1-4)GlcNAcβ(1-3)Galβ(1-4)GlcNAcβ(1-3)Galβ(1-4)GlcNAcβ(1-3)Galβ(1-4)GlcNAcβ(1-3)Galβ(1-4)GlcNAcβ(1-2)Manα(1-6)]-Manβ(1-4)GlcNAcβ(1-4)GlcNAcβ-(Lys-Val-Ala)Asn-Lys-Thr-NH2	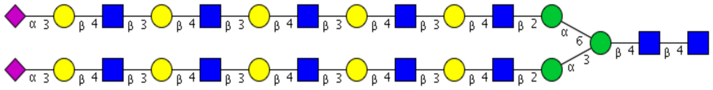
60	NeuAcα(2-3)Galβ(1-4)GlcNAcβ(1-3)Galβ(1-4)GlcNAcβ(1-3)Galβ(1-4)GlcNAcβ(1-2)Manα(1-3)[NeuAcα(2-3)Galβ(1-4)GlcNAcβ(1-3)Galβ(1-4)GlcNAcβ(1-3)Galβ(1-4)GlcNAcβ(1-2)Manα(1-6)]-Manβ(1-4)GlcNAcβ(1-4)[Fucα(1-6)]-GlcNAcβ-(Lys-Val-Ala)Asn-Lys-Thr-NH2	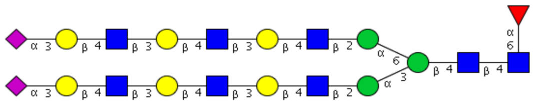
61	NeuAcα(2-3)Galβ(1-4)GlcNAcβ(1-3)Galβ(1-4)GlcNAcβ(1-3)Galβ(1-4)GlcNAcβ(1-3)Galβ(1-4)GlcNAcβ(1-2)Manα(1-3)[NeuAcα(2-3)Galβ(1-4)GlcNAcβ(1-3)Galβ(1-4)GlcNAcβ(1-3)Galβ(1-4)GlcNAcβ(1-3)Galβ(1-4)GlcNAcβ(1-2)Manα(1-6)]-Manβ(1-4)GlcNAcβ(1-4)[Fucα(1-6)]-GlcNAcβ-(Lys-Val-Ala)Asn-Lys-Thr-NH2	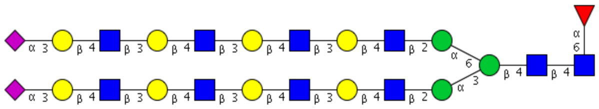
62	NeuAcα(2-3)Galβ(1-4)GlcNAcβ(1-3)Galβ(1-4)GlcNAcβ(1-2)Manα(1-3){NeuAcα(2-3)Galβ(1-4)GlcNAcβ(1-3)Galβ(1-4)GlcNAcβ(1-2)[NeuAcα(2-3)Galβ(1-4)GlcNAcβ(1-3)Galβ(1-4)GlcNAcβ(1-6)Manα(1-6)]}-Manβ(1-4)GlcNAcβ(1-4)GlcNAcβ-(Lys-Val-Ala)Asn-Lys-Thr-NH2	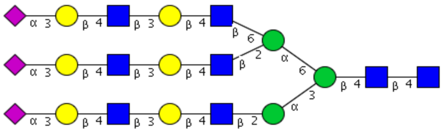
63	NeuAcα(2-3)Galβ(1-4)GlcNAcβ(1-3)Galβ(1-4)GlcNAcβ(1-3)Galβ(1-4)GlcNAcβ(1-2)Manα(1-3){NeuAcα(2-3)Galβ(1-4)GlcNAcβ(1-3)Galβ(1-4)GlcNAcβ(1-3)Galβ(1-4)GlcNAcβ(1-2)[NeuAcα(2-3)Galβ(1-4)GlcNAcβ(1-3)Galβ(1-4)GlcNAcβ(1-3)Galβ(1-4)GlcNAcβ(1-6)Manα(1-6)]}-Manβ(1-4)GlcNAcβ(1-4)GlcNAcβ-(Lys-Val-Ala)Asn-Lys-Thr-NH2	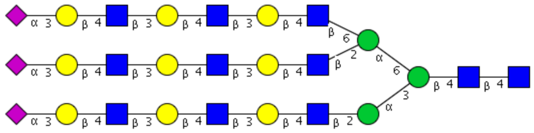

Glycan #	Common Name	Structure
64	NeuAcα(2-3)Galβ(1-4)GlcNAcβ(1-3)Galβ(1-4)GlcNAcβ(1-3)Galβ(1-4)GlcNAcβ(1-3)Galβ(1-4)GlcNAcβ(1-2)Manα(1-3){NeuAcα(2-3)Galβ(1-4)GlcNAcβ(1-3)Galβ(1-4)GlcNAcβ(1-3)Galβ(1-4)GlcNAcβ(1-3)Galβ(1-4)GlcNAcβ(1-2)[NeuAcα(2-3)Galβ(1-4)GlcNAcβ(1-3)Galβ(1-4)GlcNAcβ(1-3)Galβ(1-4)GlcNAcβ(1-3)Galβ(1-4)GlcNAcβ(1-6)Manα(1-6)]}-Manβ(1-4)GlcNAcβ(1-4)GlcNAcβ-(Lys-Val-Ala)Asn-Lys-Thr-NH2	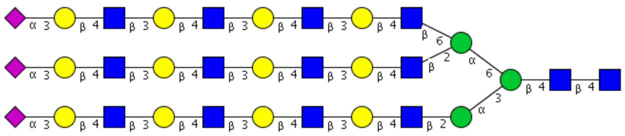
65	NeuAcα(2-3)Galβ(1-4)GlcNAcβ(1-3)Galβ(1-4)GlcNAcβ(1-2)Manα(1-3){NeuAcα(2-3)Galβ(1-4)GlcNAcβ(1-3)Galβ(1-4)GlcNAcβ(1-2)[NeuAcα(2-3)Galβ(1-4)GlcNAcβ(1-3)Galβ(1-4)GlcNAcβ(1-6)Manα(1-6)]}-Manβ(1-4)GlcNAcβ(1-4)[Fucα(1-6)]-GlcNAcβ-(Lys-Val-Ala)Asn-Lys-Thr-NH2	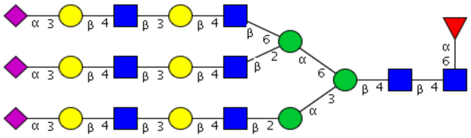
66	NeuAcα(2-3)Galβ(1-4)GlcNAcβ(1-3)Galβ(1-4)GlcNAcβ(1-3)Galβ(1-4)GlcNAcβ(1-2)Manα(1-3) -Manβ(1-4)GlcNAcβ(1-4)[Fucα(1-6)]-GlcNAcβ-(Lys-Val-Ala)Asn-Lys-Thr-NH2	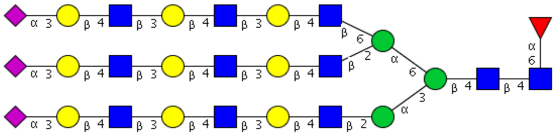
67	NeuAcα(2-3)Galβ(1-4)GlcNAcβ(1-3)Galβ(1-4)GlcNAcβ(1-3)Galβ(1-4)GlcNAcβ(1-3)Galβ(1-4)GlcNAcβ(1-2)Manα(1-3){NeuAcα(2-3)Galβ(1-4)GlcNAcβ(1-3)Galβ(1-4)GlcNAcβ(1-3)Galβ(1-4)GlcNAcβ(1-3)Galβ(1-4)GlcNAcβ(1-2)[NeuAcα(2-3)Galβ(1-4)GlcNAcβ(1-3)Galβ(1-4)GlcNAcβ(1-3)Galβ(1-4)GlcNAcβ(1-3)Galβ(1-4)GlcNAcβ(1-6)Manα(1-6)]}-Manβ(1-4)GlcNAcβ(1-4)[Fucα(1-6)]-GlcNAcβ-(Lys-Val-Ala)Asn-Lys-Thr-NH2	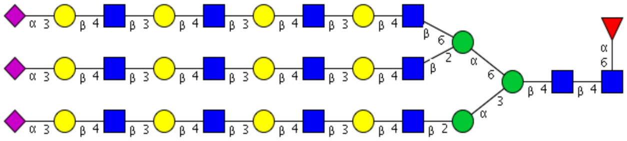
68	Gn/3'SLN/3'SLN-TriN	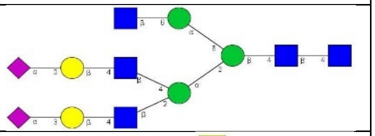
69	NeuAcα(2-3)[GalNAcβ(1-4)]-Galβ(1-4)GlcNAcβ-ethyl-NH2	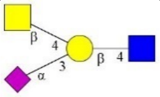
70	NeuAcα(2-3)[GalNAcβ(1-4)]-Galβ(1-4)Glcβ-ethyl-NH2	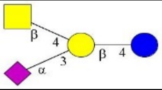
71	Galβ(1-3)GalNAcβ(1-4)[NeuAcα(2-3)]-Galβ(1-4)Glcβ-ethyl-NH2	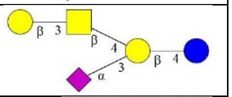
72	NeuAcα(2-3)Galβ(1-4)[Fucα(1-3)]-GlcNAcβ-propyl-NH2	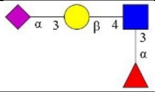

Glycan #	Common Name	Structure
73	NeuAcα(2-3)Galβ(1-3)[Fucα(1-4)]-GlcNAcβ(1-3)Galβ(1-4)[Fucα(1-3)]-GlcNAcβ-ethyl-NH2	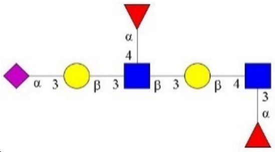
74	NeuAcα(2-3)Galβ(1-4)[Fucα(1-3)]-GlcNAcβ(1-3)Galβ(1-4)[Fucα(1-3)]-GlcNAcβ-ethyl-NH2	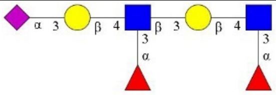
75	NeuAcα(2-3)Galβ(1-4)[Fucα(1-3)]-GlcNAcβ(1-3)Galβ(1-4)[Fucα(1-3)]-GlcNAcβ(1-3)Galβ(1-4)[Fucα(1-3)]-GlcNAcβ-ethyl-NH2	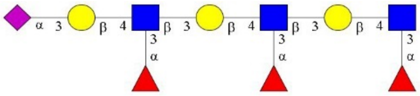
76	NeuAcα(2-3)Galβ(1-4)[Fucα(1-3)]-GlcNAcβ(1-3)Galβ(1-4)[Fucα(1-3)]-GlcNAcβ(1-3)Galβ(1-4)[Fucα(1-3)]-GlcNAcβ(1-3)Galβ(1-3)GalNAcα-Thr-NH2	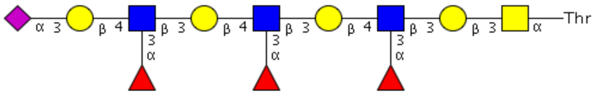
77	NeuAcα(2-3)Galβ(1-4)[Fucα(1-3)]-GlcNAcβ(1-3)Galβ(1-4)[Fucα(1-3)]-GlcNAcβ(1-3)Galβ(1-4)[Fucα(1-3)]-GlcNAcβ(1-3)GalNAcα-Thr-NH2	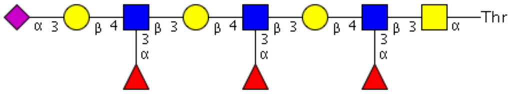
78	NeuAcα(2-3)Galβ(1-4)[Fucα(1-3)]-GlcNAcβ(1-3)Galβ(1-4)[Fucα(1-3)]-GlcNAcβ(1-3)Galβ(1-4)[Fucα(1-3)]-GlcNAcβ(1-3)[NeuAcα(2-3)Galβ(1-4)[Fucα(1-3)]-GlcNAcβ(1-3)Galβ(1-4)[Fucα(1-3)]-GlcNAcβ(1-3)Galβ(1-4)[Fucα(1-3)]-GlcNAcβ(1-6)]-GalNAcα-Thr-NH2	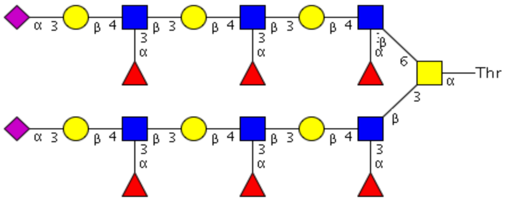
79	NeuAcα(2-3)Galβ(1-4)[Fucα(1-3)]-GlcNAcβ(1-2)Manα(1-3)[NeuAcα(2-3)Galβ(1-4)[Fucα(1-3)]-GlcNAcβ(1-2)Manα(1-6)]-Manβ(1-4)GlcNAcβ(1-4)GlcNAcβ-(Lys-Val-Ala)Asn-(Lys-Thr)NH2	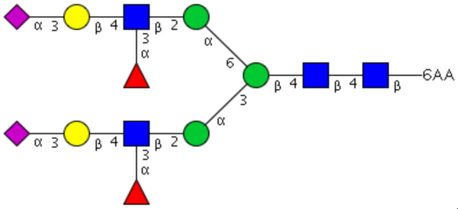
80	NeuAcα(2-6)Galb(1-4)(6S)GlcNacβ-ethyl-NH2	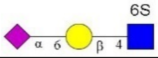
81	NeuAcα(2-6)Galβ(1-4)6-O-sulfo-GlcNAcβ-propyl-NH2	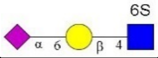
82	NeuAcα(2-6)Galβ(1-4)Glcβ-ethyl-NH2	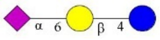
83	NeuAcα(2-6)Galβ(1-4)GlcNAcβ-ethyl-NH2	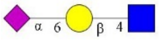
84	NeuAcα(2-6)Galβ(1-4)GlcNAcβ(1-3)Galβ(1-4)GlcNAcβ-ethyl-NH2	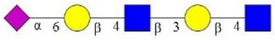
85	NeuAcα(2-6)Galβ(1-4)GlcNAcβ(1-3)Galβ(1-4)GlcNAcβ(1-3)Galβ(1-4)GlcNAcβ-ethyl-NH2	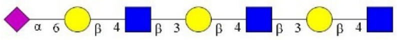
86	NeuAcα(2-6)GalNAcβ(1-4)GlcNAcβ-ethyl-NH2	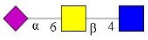
87	NeuAcα(2-6)Galβ(1-4)GlcNAcβ(1-3)Galβ(1-3)GalNAcα-Thr-NH2	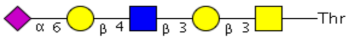
88	NeuAcα(2-6)Galβ(1-4)GlcNAcβ(1-3)Galβ(1-4)GlcNAcβ(1-3)Galβ(1-3)GalNAcα-Thr-NH2	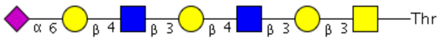
89	NeuAcα(2-6)Galβ(1-4)GlcNAcβ(1-3)Galβ(1-4)GlcNAcβ(1-3)Galβ(1-4)GlcNAcβ(1-3)Galβ(1-3)GalNAcα-Thr-NH2	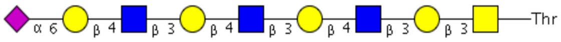

Glycan #	Common Name	Structure
90	NeuAcα(2-6)Galβ(1-4)GlcNAcβ(1-3)Galβ(1-4)GlcNAcβ(1-3)Galβ(1-4)GlcNAcβ(1-3)Galβ(1-4)GlcNAcβ(1-3)Galβ(1-3)GalNAcα-Thr-NH2	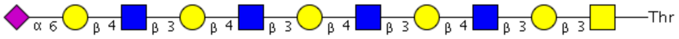
91	NeuAcα(2-6)Galβ(1-4)GlcNAcβ(1-3)Galβ(1-4)GlcNAcβ(1-3)Galβ(1-4)GlcNAcβ(1-3)Galβ(1-4)GlcNAcβ(1-3)Galβ(1-4)GlcNAcβ(1-3)Galβ(1-3)GalNAcα-Thr-NH2	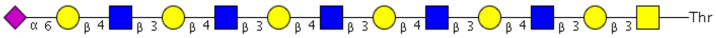
92	NeuAcα(2-6)Galβ(1-4)GlcNAcβ(1-6)[Galβ(1-3)]-GalNAcα-Thr-NH2	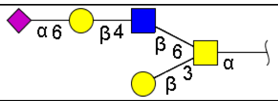
93	NeuAcα(2-6)Galβ(1-4)GlcNAcβ(1-3)Galβ(1-4)GlcNAcβ(1-6)[Galβ(1-3)]-GalNAcα-Thr-NH2	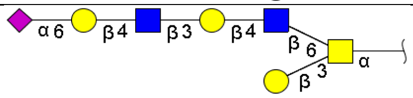
94	NeuAcα(2-6)Galβ(1-4)GlcNAcβ(1-3)Galβ(1-4)GlcNAcβ(1-3)Galβ(1-4)GlcNAcβ(1-6)[Galβ(1-3)]-GalNAcα-Thr-NH2	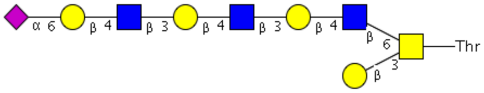
95	NeuAcα(2-6)Galβ(1-4)GlcNAcβ(1-3)Galβ(1-4)GlcNAcβ(1-3)Galβ(1-4)GlcNAcβ(1-3)Galβ(1-4)GlcNAcβ(1-6)[Galβ(1-3)]-GalNAcα-Thr-NH2	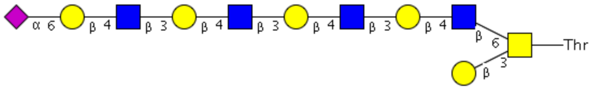
96	NeuAcα(2-6)Galβ(1-4)GlcNAcβ(1-3)Galβ(1-4)GlcNAcβ(1-3)Galβ(1-4)GlcNAcβ(1-3)Galβ(1-4)GlcNAcβ(1-3)Galβ(1-4)GlcNAcβ(1-6)[Galβ(1-3)]-GalNAcα-Thr-NH2	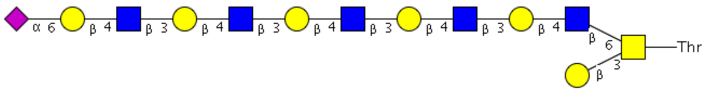
97	NeuAcα(2-6)Galβ(1-4)GlcNAcβ(1-3)Galβ(1-4)GlcNAcβ(1-3)Galβ(1-4)GlcNAcβ(1-3)Galβ(1-4)GlcNAcβ(1-6)[NeuAcα(2-6)Galβ(1-4)GlcNAcβ(1-3)Galβ(1-4)GlcNAcβ(1-3)Galβ(1-4)GlcNAcβ(1-3)Galβ(1-3)]-GalNAcα-Thr-NH2	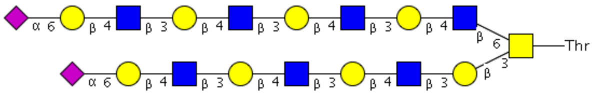
98	NeuAcα(2-6)Galβ(1-4)GlcNAcβ(1-3)Galβ(1-4)GlcNAcβ(1-3)Galβ(1-4)GlcNAcβ(1-3)Galβ(1-4)GlcNAcβ(1-3)Galβ(1-4)GlcNAcβ(1-6)[NeuAcα(2-6)Galβ(1-4)GlcNAcβ(1-3)Galβ(1-4)GlcNAcβ(1-3)Galβ(1-4)GlcNAcβ(1-3)Galβ(1-4)GlcNAcβ(1-3)Galβ(1-3)]-GalNAcα-Thr-NH2	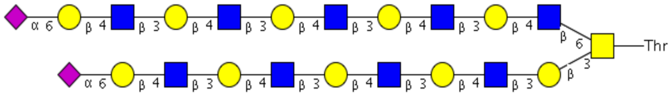
99	NeuAcα(2-6)Galβ(1-4)GlcNAcβ(1-3)GalNAcα-Thr-NH2	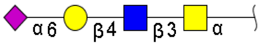
100	NeuAcα(2-6)Galβ(1-4)GlcNAcβ(1-3)Galβ(1-4)GlcNAcβ(1-3)GalNAcα-Thr-NH2	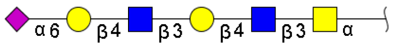
101	NeuAcα(2-6)Galβ(1-4)GlcNAcβ(1-3)Galβ(1-4)GlcNAcβ(1-3)Galβ(1-4)GlcNAcβ(1-3)GalNAcα-Thr-NH2	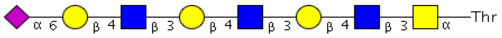
102	NeuAcα(2-6)Galβ(1-4)GlcNAcβ(1-3)Galβ(1-4)GlcNAcβ(1-3)Galβ(1-4)GlcNAcβ(1-3)Galβ(1-4)GlcNAcβ(1-3)GalNAcα-Thr-NH2	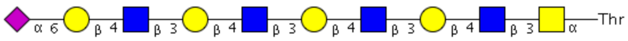

Glycan #	Common Name	Structure
103	NeuAcα(2-6)Galβ(1-4)GlcNAcβ(1-3)Galβ(1-4)GlcNAcβ(1-3)Galβ(1-4)GlcNAcβ(1-3)Galβ(1-4)GlcNAcβ(1-3)Galβ(1-4)GlcNAcβ(1-3)GalNAcα-Thr-NH2	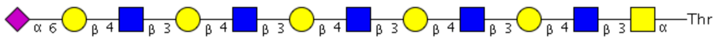
104	NeuAcα(2-6)Galβ(1-4)GlcNAcβ(1-3)[NeuAcα(2-6)Galβ(1-4)GlcNAcβ(1-6)]-GalNAcα-Thr-NH2	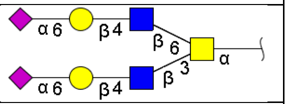
105	NeuAcα(2-6)Galβ(1-4)GlcNAcβ(1-3)Galβ(1-4)GlcNAcβ(1-3)[NeuAcα(2-6)Galβ(1-4)GlcNAcβ(1-3)Galβ(1-4)GlcNAcβ(1-6)]-GalNAcα-Thr-NH2	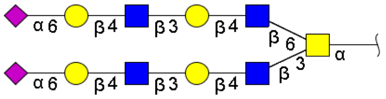
106	NeuAcα(2-6)Galβ(1-4)GlcNAcβ(1-3)Galβ(1-4)GlcNAcβ(1-3)Galβ(1-4)GlcNAcβ(1-3)[NeuAcα(2-6)Galβ(1-4)GlcNAcβ(1-3)Galβ(1-4)GlcNAcβ(1-3)Galβ(1-4)GlcNAcβ(1-6)]-GalNAcα-Thr-NH2	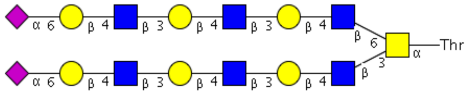
107	NeuAcα(2-6)Galβ(1-4)GlcNAcβ(1-3)Galβ(1-4)GlcNAcβ(1-3)Galβ(1-4)GlcNAcβ(1-3)Galβ(1-4)GlcNAcβ(1-3)[NeuAcα(2-6)Galβ(1-4)GlcNAcβ(1-3)Galβ(1-4)GlcNAcβ(1-3)Galβ(1-4)GlcNAcβ(1-3)Galβ(1-4)GlcNAcβ(1-6)]-GalNAcα-Thr-NH2	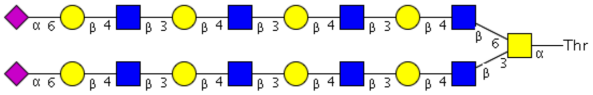
108	NeuAcα(2-6)Galβ(1-4)GlcNAcβ(1-3)Galβ(1-4)GlcNAcβ(1-3)Galβ(1-4)GlcNAcβ(1-3)Galβ(1-4)GlcNAcβ(1-3)Galβ(1-4)GlcNAcβ(1-3)[NeuAcα(2-6)Galβ(1-4)GlcNAcβ(1-3)Galβ(1-4)GlcNAcβ(1-3)Galβ(1-4)GlcNAcβ(1-3)Galβ(1-4)GlcNAcβ(1-3)Galβ(1-4)GlcNAcβ(1-6)]-GalNAcα-Thr-NH2	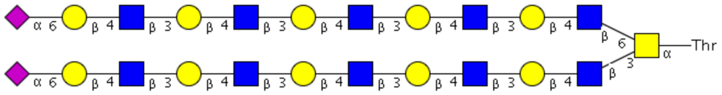
109	NeuAcα(2-6)Galβ(1-4)GlcNAcβ(1-3)Galβ(1-4)GlcNAcβ(1-3)Galβ(1-4)GlcNAcβ(1-3)Galβ(1-4)GlcNAcβ(1-6)GalNAcα-Thr-NH2	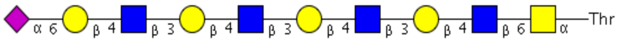
110	NeuAcα(2-6)Galβ(1-4)GlcNAcβ(1-3)Galβ(1-4)GlcNAcβ(1-3)Galβ(1-4)GlcNAcβ(1-3)Galβ(1-4)GlcNAcβ(1-3)Galβ(1-4)GlcNAcβ(1-6)GalNAcα-Thr-NH2	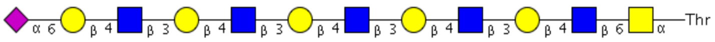
111	NeuAcα(2-6)Galβ(1-4)GlcNAcβ(1-3)Galβ(1-4)GlcNAcβ(1-3)Galβ(1-4)GlcNAcβ(1-6)[NeuAcα(2-6)Galβ(1-4)GlcNAcβ(1-3)Galβ(1-4)GlcNAcβ(1-3)] Galβ(1-4)GlcNAcβ-ethyl-NH2	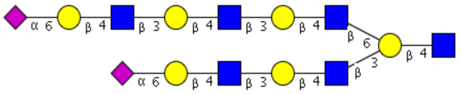
112	NeuAcα(2-6)Galβ(1-3)GlcNAcβ(1-3)Galβ(1-4)GlcNAcβ(1-6)[NeuAcα(2-6)Galβ(1-3)GlcNAcβ(1-3)] Galβ(1-4)GlcNAcβ-ethyl-NH2	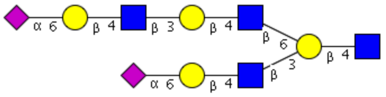
113	Galβ(1-4)GlcNAcβ(1-2)Manα(1-3)[NeuAcα(2-6)Galβ(1-4)GlcNAcβ(1-2)Manα(1-6)]-Manβ(1-4)GlcNAcβ(1-4)GlcNAcβ-Asn-NH2	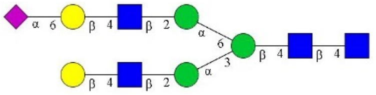
114	NeuAcα(2-6)Galβ(1-4)GlcNAcβ(1-2)Manα(1-3)[Galβ(1-4)GlcNAcβ(1-2)Manα(1-6)]-Manβ(1-4)GlcNAcβ(1-4)GlcNAcβ-Asn-NH2	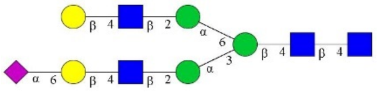

Glycan #	Common Name	Structure
115	GlcNAcβ(1-2)Manα(1-3)[NeuAcα(2-6)Galβ(1-4)GlcNAcβ(1-2)Manα(1-6)]-Manβ(1-4)GlcNAcβ(1-4)GlcNAcβ-Asn-NH2	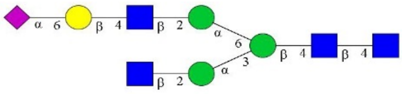
116	NeuAcα(2-6)Galβ(1-4)GlcNAcβ(1-2)Manα(1-3)[NeuAcα(2-6)Galβ(1-4)GlcNAcβ(1-2)Manα(1-6)]-Manβ(1-4)GlcNAcβ(1-4)GlcNAcβ-Asn-NH2	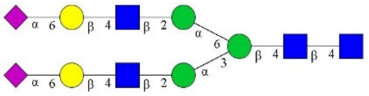
117	NeuAcα(2-6)Galβ(1-4)GlcNAcβ(1-3)Galβ(1-4)GlcNAcβ(1-2)Manα(1-3)[NeuAcα(2-6)Galβ(1-4)GlcNAcβ(1-3)Galβ(1-4)GlcNAcβ(1-2)Manα(1-6)]-Manβ(1-4)GlcNAcβ(1-4)GlcNAcβ-Asn-NH2	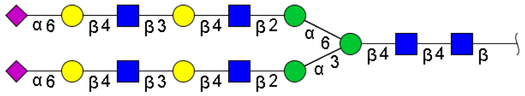
118	NeuAcα(2-6)Galβ(1-4)GlcNAcβ(1-3)Galβ(1-4)GlcNAcβ(1-2)Manα(1-3)[NeuAcα(2-6)Galβ(1-4)GlcNAcβ(1-3)Galβ(1-4)GlcNAcβ(1-2)Manα(1-6)]-Manβ(1-4)GlcNAcβ(1-4)GlcNAcβ-(Lys-Val-Ala)Asn-Lys-Thr-NH2	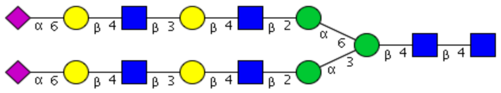
119	NeuAcα(2-6)Galβ(1-4)GlcNAcβ(1-3)Galβ(1-4)GlcNAcβ(1-3)Galβ(1-4)GlcNAcβ(1-2)Manα(1-3)[NeuAcα(2-6)Galβ(1-4)GlcNAcβ(1-3)Galβ(1-4)GlcNAcβ(1-3)Galβ(1-4)GlcNAcβ(1-2)Manα(1-6)]-Manβ(1-4)GlcNAcβ(1-4)GlcNAcβ-Asn-NH2	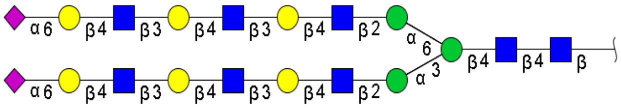
120	NeuAcα(2-6)Galβ(1-4)GlcNAcβ(1-3)Galβ(1-4)GlcNAcβ(1-3)Galβ(1-4)GlcNAcβ(1-2)Manα(1-3)[NeuAcα(2-6)Galβ(1-4)GlcNAcβ(1-3)Galβ(1-4)GlcNAcβ(1-3)Galβ(1-4)GlcNAcβ(1-2)Manα(1-6)]-Manβ(1-4)GlcNAcβ(1-4)GlcNAcβ-(Lys-Val-Ala)Asn-Lys-Thr-NH2	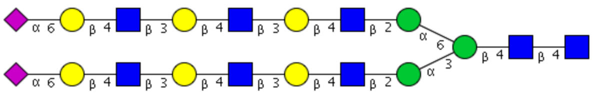
121	NeuAcα(2-6)Galβ(1-4)GlcNAcβ(1-3)Galβ(1-4)GlcNAcβ(1-3)Galβ(1-4)GlcNAcβ(1-3)Galβ(1-4)GlcNAcβ(1-2)Manα(1-3)[NeuAcα(2-6)Galβ(1-4)GlcNAcβ(1-3)Galβ(1-4)GlcNAcβ(1-3)Galβ(1-4)GlcNAcβ(1-3)Galβ(1-4)GlcNAcβ(1-2)Manα(1-6)]-Manβ(1-4)GlcNAcβ(1-4)GlcNAcβ-(Lys-Val-Ala)Asn-Lys-Thr-NH2	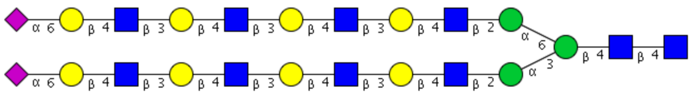
122	NeuAcα(2-6)Galβ(1-4)GlcNAcβ(1-3)Galβ(1-4)GlcNAcβ(1-3)Galβ(1-4)GlcNAcβ(1-3)Galβ(1-4)GlcNAcβ(1-3)Galβ(1-4)GlcNAcβ(1-2)Manα(1-3)[NeuAcα(2-6)Galβ(1-4)GlcNAcβ(1-3)Galβ(1-4)GlcNAcβ(1-3)Galβ(1-4)GlcNAcβ(1-3)Galβ(1-4)GlcNAcβ(1-3)Galβ(1-4)GlcNAcβ(1-2)Manα(1-6)]-Manβ(1-4)GlcNAcβ(1-4)GlcNAcβ-(Lys-Val-Ala)Asn-Lys-Thr-NH2	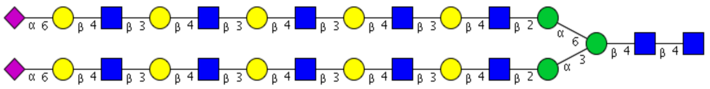

Glycan #	Common Name	Structure
123	NeuAcα(2-6)Galβ(1-4)GlcNAcβ(1-3)Galβ(1-4)GlcNAcβ(1-2)Manα(1-3)[NeuAcα(2-6)Galβ(1-4)GlcNAcβ(1-3)Galβ(1-4)GlcNAcβ(1-2)Manα(1-6)]-Manβ(1-4)GlcNAcβ(1-4)[Fucα(1-6)]-GlcNAcβ-(Lys-Val-Ala)Asn-Lys-Thr-NH2	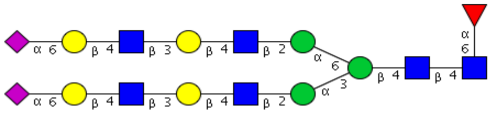
124	NeuAcα(2-6)Galβ(1-4)GlcNAcβ(1-3)Galβ(1-4)GlcNAcβ(1-3)Galβ(1-4)GlcNAcβ(1-2)Manα(1-3)[NeuAcα(2-6)Galβ(1-4)GlcNAcβ(1-3)Galβ(1-4)GlcNAcβ(1-3)Galβ(1-4)GlcNAcβ(1-2)Manα(1-6)]-Manβ(1-4)GlcNAcβ(1-4)[Fucα(1-6)]-GlcNAcβ-(Lys-Val-Ala)Asn-Lys-Thr-NH2	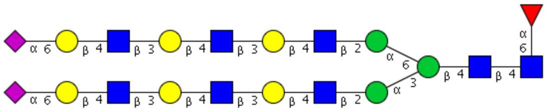
125	NeuAcα(2-6)Galβ(1-4)GlcNAcβ(1-3)Galβ(1-4)GlcNAcβ(1-3)Galβ(1-4)GlcNAcβ(1-3)Galβ(1-4)GlcNAcβ(1-2)Manα(1-3)[NeuAcα(2-6)Galβ(1-4)GlcNAcβ(1-3)Galβ(1-4)GlcNAcβ(1-3)Galβ(1-4)GlcNAcβ(1-3)Galβ(1-4)GlcNAcβ(1-2)Manα(1-6)]-Manβ(1-4)GlcNAcβ(1-4)[Fucα(1-6)]-GlcNAcβ-(Lys-Val-Ala)Asn-Lys-Thr-NH2	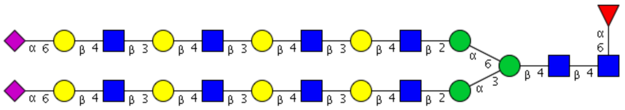
126	NeuAcα(2-6)Galβ(1-4)GlcNAcβ(1-3)Galβ(1-4)GlcNAcβ(1-2)Manα(1-3){NeuAcα(2-6)Galβ(1-4)GlcNAcβ(1-3)Galβ(1-4)GlcNAcβ(1-2)[NeuAcα(2-6)Galβ(1-4)GlcNAcβ(1-3)Galβ(1-4)GlcNAcβ(1-6)Manα(1-6)]}-Manβ(1-4)GlcNAcβ(1-4)GlcNAcβ-(Lys-Val-Ala)Asn-Lys-Thr-NH2	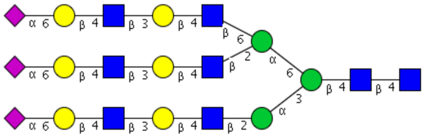
127	NeuAcα(2-6)Galβ(1-4)GlcNAcβ(1-3)Galβ(1-4)GlcNAcβ(1-3)Galβ(1-4)GlcNAcβ(1-2)Manα(1-3){NeuAcα(2-6)Galβ(1-4)GlcNAcβ(1-3)Galβ(1-4)GlcNAcβ(1-3)Galβ(1-4)GlcNAcβ(1-2)[NeuAcα(2-6)Galβ(1-4)GlcNAcβ(1-3)Galβ(1-4)GlcNAcβ(1-3)Galβ(1-4)GlcNAcβ(1-6)Manα(1-6)]}-Manβ(1-4)GlcNAcβ(1-4)GlcNAcβ-(Lys-Val-Ala)Asn-Lys-Thr-NH2	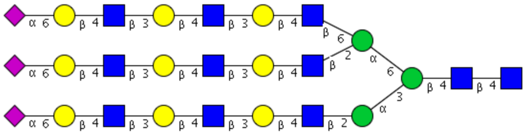
128	NeuAcα(2-6)Galβ(1-4)GlcNAcβ(1-3)Galβ(1-4)GlcNAcβ(1-3)Galβ(1-4)GlcNAcβ(1-3)Galβ(1-4)GlcNAcβ(1-2)Manα(1-3){NeuAcα(2-6)Galβ(1-4)GlcNAcβ(1-3)Galβ(1-4)GlcNAcβ(1-3)Galβ(1-4)GlcNAcβ(1-3)Galβ(1-4)GlcNAcβ(1-2)[NeuAcα(2-6)Galβ(1-4)GlcNAcβ(1-3)Galβ(1-4)GlcNAcβ(1-3)Galβ(1-4)GlcNAcβ(1-3)Galβ(1-4)GlcNAcβ(1-6)Manα(1-6)]}-Manβ(1-4)GlcNAcβ(1-4)GlcNAcβ-(Lys-Val-Ala)Asn-Lys-Thr-NH2	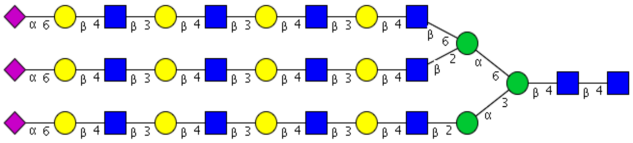

Glycan #	Common Name	Structure
129	NeuAcα(2-6)Galβ(1-4)GlcNAcβ(1-3)Galβ(1-4)GlcNAcβ(1-2)Manα(1-3){NeuAcα(2-6)Galβ(1-4)GlcNAcβ(1-3)Galβ(1-4)GlcNAcβ(1-2)[NeuAcα(2-6)Galβ(1-4)GlcNAcβ(1-3)Galβ(1-4)GlcNAcβ(1-6)Manα(1-6)]}-Manβ(1-4)GlcNAcβ(1-4)[Fucα(1-6)]-GlcNAcβ-(Lys-Val-Ala)Asn-Lys-Thr-NH2	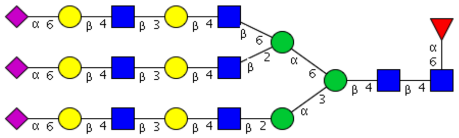
130	NeuAcα(2-6)Galβ(1-4)GlcNAcβ(1-3)Galβ(1-4)GlcNAcβ(1-3)Galβ(1-4)GlcNAcβ(1-2)Manα(1-3){NeuAcα(2-6)Galβ(1-4)GlcNAcβ(1-3)Galβ(1-4)GlcNAcβ(1-3)Galβ(1-4)GlcNAcβ(1-2)[NeuAcα(2-6)Galβ(1-4)GlcNAcβ(1-3)Galβ(1-4)GlcNAcβ(1-3)Galβ(1-4)GlcNAcβ(1-6)Manα(1-6)]}-Manβ(1-4)GlcNAcβ(1-4)[Fucα(1-6)]-GlcNAcβ-(Lys-Val-Ala)Asn-Lys-Thr-NH2	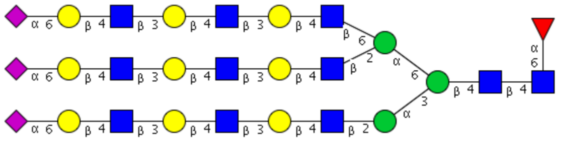
131	NeuAcα(2-6)Galβ(1-4)GlcNAcβ(1-3)Galβ(1-4)GlcNAcβ(1-3)Galβ(1-4)GlcNAcβ(1-3)Galβ(1-4)GlcNAcβ(1-2)Manα(1-3){NeuAcα(2-6)Galβ(1-4)GlcNAcβ(1-3)Galβ(1-4)GlcNAcβ(1-3)Galβ(1-4)GlcNAcβ(1-3)Galβ(1-4)GlcNAcβ(1-2)[NeuAcα(2-6)Galβ(1-4)GlcNAcβ(1-3)Galβ(1-4)GlcNAcβ(1-3)Galβ(1-4)GlcNAcβ(1-3)Galβ(1-4)GlcNAcβ(1-6)Manα(1-6)]}-Manβ(1-4)GlcNAcβ(1-4)[Fucα(1-6)]-GlcNAcβ-(Lys-Val-Ala)Asn-Lys-Thr-NH2	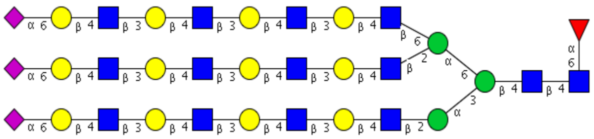
132	LN/6'SLN/6'SLN-TriN	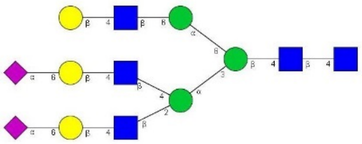
133	6'SLNLN/6'SLN/6'SLN-TriN	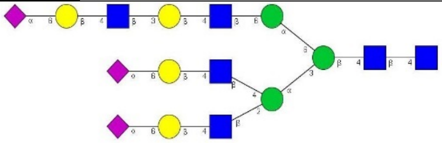
134	6'SLN/LeX/LeX-TriN	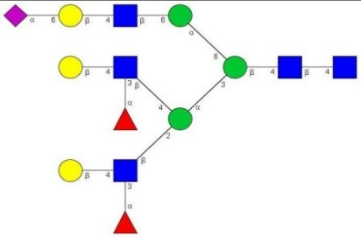

Glycan #	Common Name	Structure
135	6'SLNLN/LeX/LeX-TriN	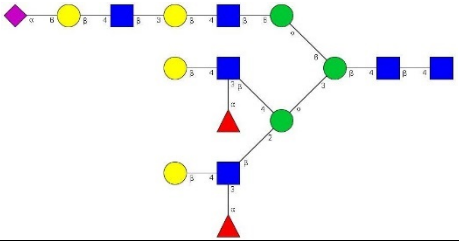

Table S2. Data Collection and Refinement Statistics for H6 HA G225D and Receptor Analog Complexes

Data collection	Apo	6′-SLN	LSTa	3′-SLN
Beamline	SSRL11-1	APS-23	APS-23	SSRL11-1
Wavelength (Å)	0.97945	1.03320	1.03320	0.97945
Space Group	C2	C2	C2	C2
Unit cell parameters (Å,°)	a=185.0, b=98.3, c=133.2 β=126.4°	a=185.5, b=98.9, c=133.6 β=126.6°	a=185.1, b=99.2, c=133.3 β=126.3°	a=185.0, b=98.7, c=132.7 β=126.7°
Resolution range (Å)	50.00-2.19 (2.23-2.19)[a]	50.00-2.00 (2.03-2.00)	50.00-2.11 (2.15-2.11)	50.00-2.91 (2.91-2.86)
Observations	359,608	403,653	354,219	160,004
Unique refs.	96,720 (4680)	126,685 (6125)	113,368 (5393)	43,053 (2115)
Completeness (%)	98.5 (95.7)	97.5 (94.9)	99.2 (94.7)	97.5 (95.6)
I/σ(I)	19.1 (2.3)	16.2 (1.3)	18.3 (2.4)	14.9 (3.3)
$CC_{1/2}$[b]	0.97 (0.91)	0.86 (0.53)	0.94 (0.83)	0.97 (0.95)
R_{sym}[c]	0.07 (0.39)	0.11 (0.81)	0.10 (0.41)	0.08 (0.28)
R_{pim}[d]	0.03 (0.20)	0.06 (0.44)	0.05 (0.25)	0.04 (0.14)
Redundancy	3.7 (3.3)	3.2 (3.0)	3.1 (2.3)	3.7 (3.6)
Refinement Statistics				
Resolution (Å)	50.00-2.19 (2.23-2.19)	50.00-2.00 (2.03-2.00)	50.00-2.11 (2.15-2.11)	50.00-2.86 (2.86-2.91)
No. reflections	96,671	126,567	113,339	43,022
R_{cryst}[e]/R_{free}[f]	0.213/0.251	0.212/0.246	0.213/0.254	0.208/0.244
No. atoms				
Protein	11909	11909	11910	11910
Carbohydrate	56	116	89	130
Water	410	582	479	87
Wilson B ($Å^2$)	37	35	35	44
Av. B value ($Å^2$)				
Overall	45	41	41	38
Ligand	-	77	66	69
R.m.s deviation from ideal geometry				
Bond length (Å)	0.012	0.012	0.012	0.010
Bond angle (º)	1.5	1.5	1.4	1.3
Ramachandran Plot (%)[g]				
Favored	97.9	97.8	97.6	96.2
Outliers	0	0.1	0.1	0.1
PDB ID	5T08	5T0B	5T0E	5T0D

[a]Parentheses refer to outer shell statistics.
[b]$CC_{1/2}$ = Pearson Correlation Coefficient between two random half datasets.
[c]$R_{sym} = \Sigma_{hkl} \Sigma_i | I_{hkl,i} - <I_{hkl}> | / \Sigma_{hkl} \Sigma_i I_{hkl,i}$, where $I_{hkl,i}$ is the scaled intensity of the ith measurement of reflection *h, k, l*, and $<I_{hkl}>$ is the average intensity for that reflection.
[d]$R_{pim} = \Sigma_{hkl} (1/(n-1))^{1/2} \Sigma_i | I_{hkl,i} - <I_{hkl}> | / \Sigma_{hkl} \Sigma_i I_{hkl,i}$, where n is the redundancy.
[e]$R_{cryst} = \Sigma_{hkl} | F_o - F_c | / \Sigma_{hkl} | F_o |$ x 100, where F_o and F_c are the observed and calculated structures factors.
[f]R_{free} was calculated as for R_{cryst}, but on a test set of 5% of the data excluded from refinement.
[g]Calculated using MolProbity (Davis et al, 2007)

Table S3. Top hits of A/Taiwan/2/13 and A/Taiwan/2/13 G225D with the structures shown. # denotes hit number, with corresponding RFU.

A/Taiwan/2/13 H6N1	A/Vietnam/1203/04 H5N1	G225D	A/HK/6983/10 H3N2	Structure
#1 22017	#5 20193	87	-5	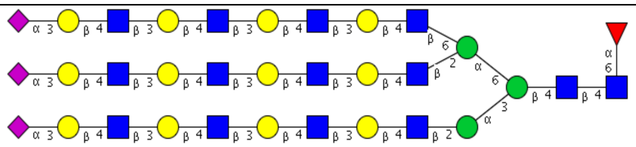
#2 21690	#7 13360	-18	17	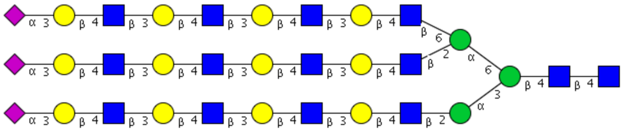
#3 13033	7504	-8	1	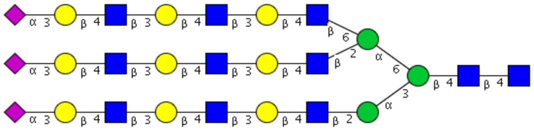

G225D	A/HK/6983/10 H3N2	A/Vietnam/1203/04 H5N1	A/Taiwan/2/13 H6N1	Structure
#1 27357	#9 5278	20	1157	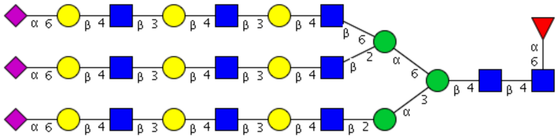
#3 12497	#1 20209	20	1270	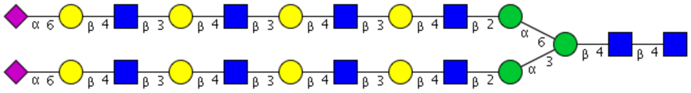
#6 6887	#4 11550	8	60	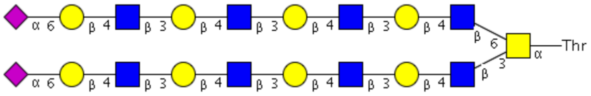

Table S4. Apparent avidities of the A/Taiwan/2/13 and the G225D mutant

Binding of A/Taiwan/2/2013 and G225D variant HA to biotinylated α2-3 and α2-6 N-glycan receptors (quoted values are K_d apparent (μg/mL), ± standard error):

	**A/Taiwan/2/2013**	**G225D**
**3SLN**	14.31± 1.22[#]	n/b*
**$3SLN_2$**	17.12 ± 1.84[#]	n/b*
**$3SLN_3$**	2.93 ± 0.14	n/b*
**$3SLN_4$**	1.02 ± 0.08	n/b*
**6SLN**	n/b*	n/b*
**$6SLN_2$**	n/b*	n/b*
**$6SLN_3$**	44.67 ± 7.04[#]	n/b*
**$6SLN_4$**	5.58 ± 0.38	43.88 ± 6.98[#]

* n/b, no binding/insufficient to estimate K_d.
[#] K_d estimated via extrapolation to maximal binding.

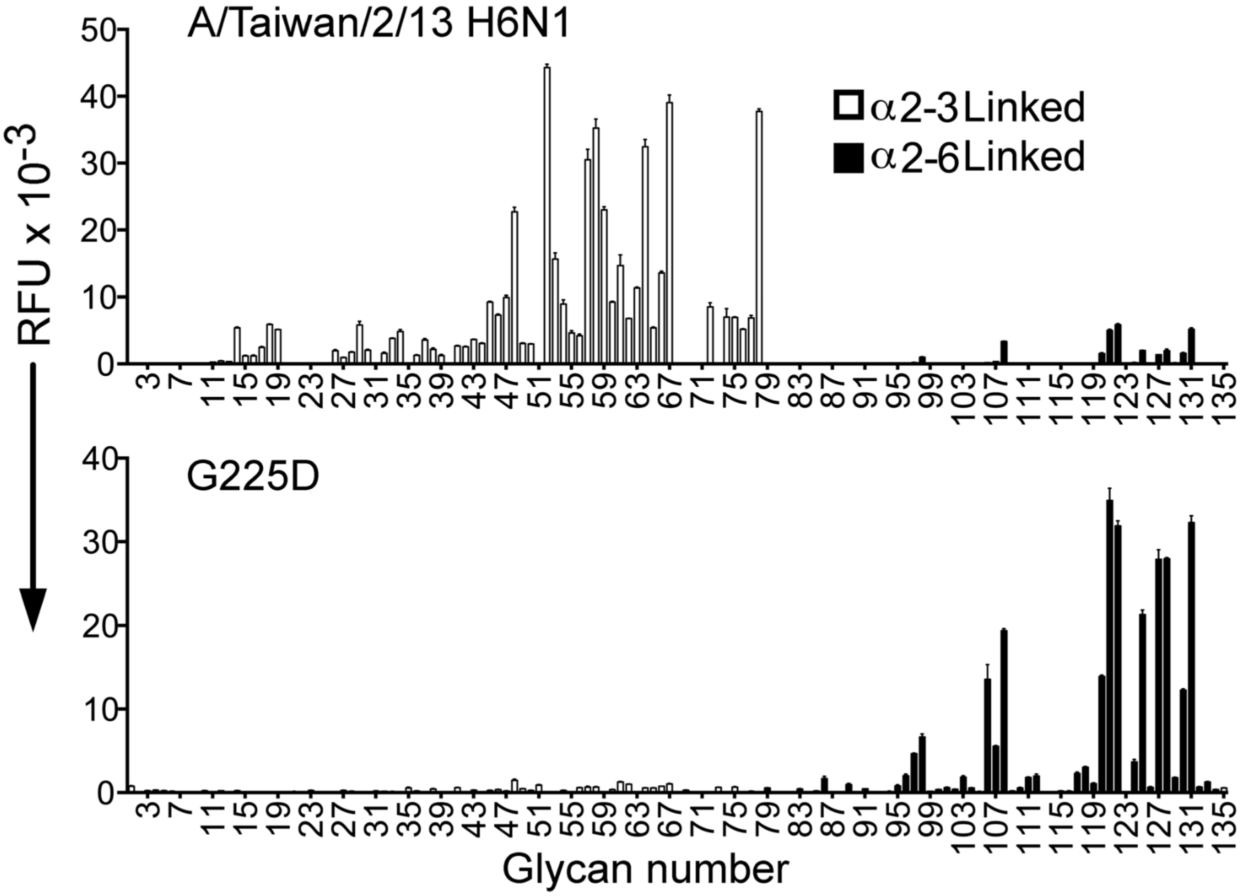

Figure S1. Receptor binding specificity of H6N1 wild-type and G225D mutant HAs expressed in insect cells. Glycan microarray analysis of recombinant H6 HA's from insect cells indicates specific binding to α2-3 sialosides for the wild type and specific binding to α2-6 sialosides for the G225D mutant.

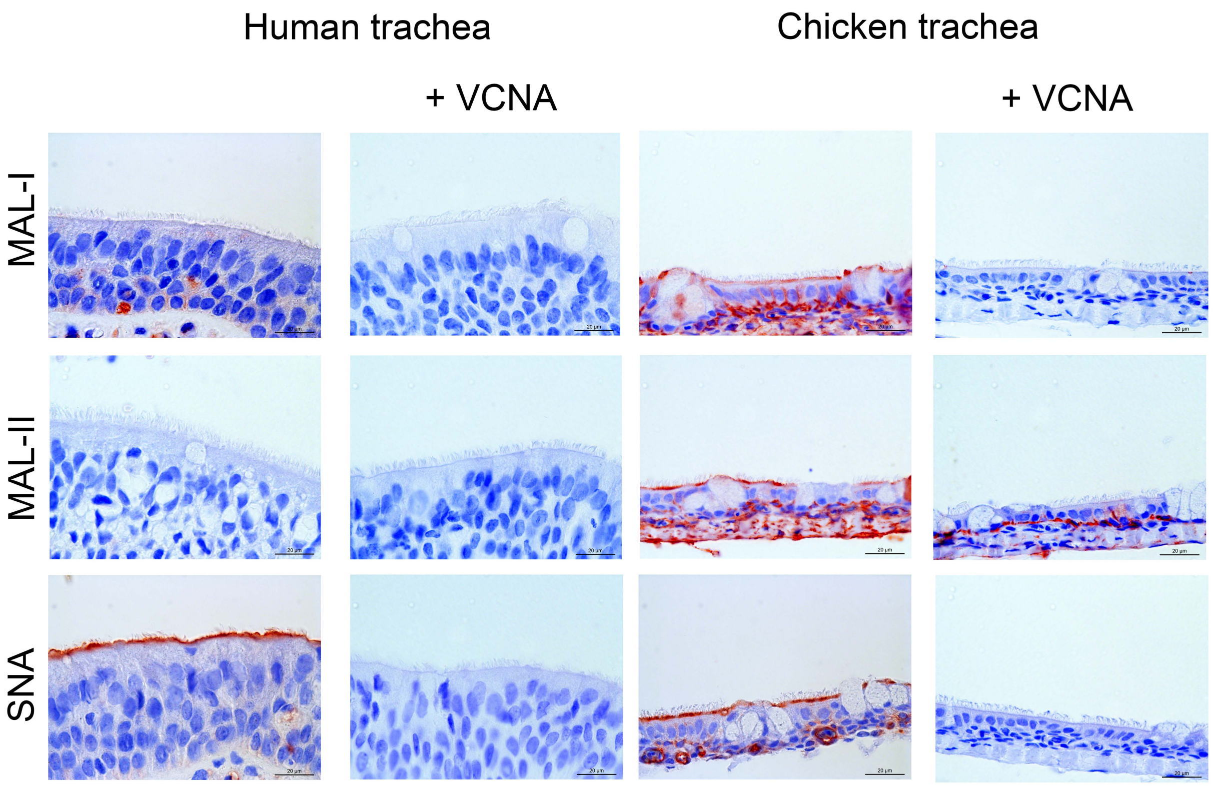

Figure S2. Analysis of plant lectin binding to chicken and human trachea epithelial cells. (**A**) Binding of biotinylated MAL-1, MAL-II and SNA lectins to chicken and human tracheal tissue, including neuraminidase treatments to remove all sialic acids. Binding is detected using streptavidin-HRP and developed with AEC.

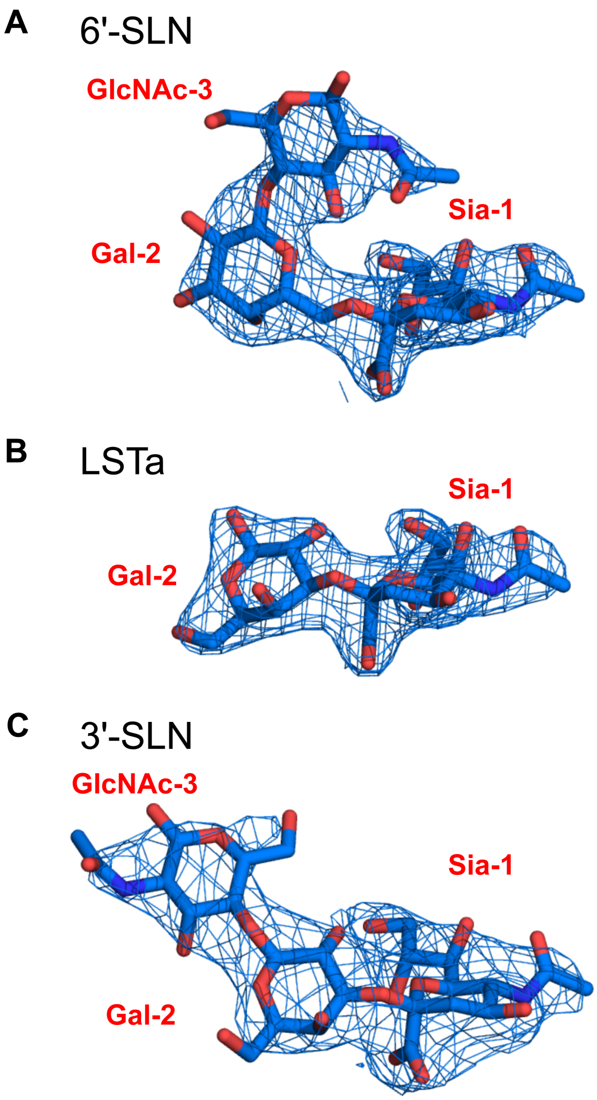

Figure S3. Electron density for receptor analogs in the H6 G225D HA crystal structures. (**A-C**) Electron density maps (2Fo-Fc) of human receptor analog 6’-sialyl-N-acetyl-lactososamine (6′-SLN) (A) and avian receptor analogues 3’-sialyl-lacto-n-tetraose, LSTa (B) and 3’-sialyl-N-acetyl-lactososamine 3′-SLN (**C**) in crystal structures of Taiwan2 H6 G225D HA contoured at a 1σ level.

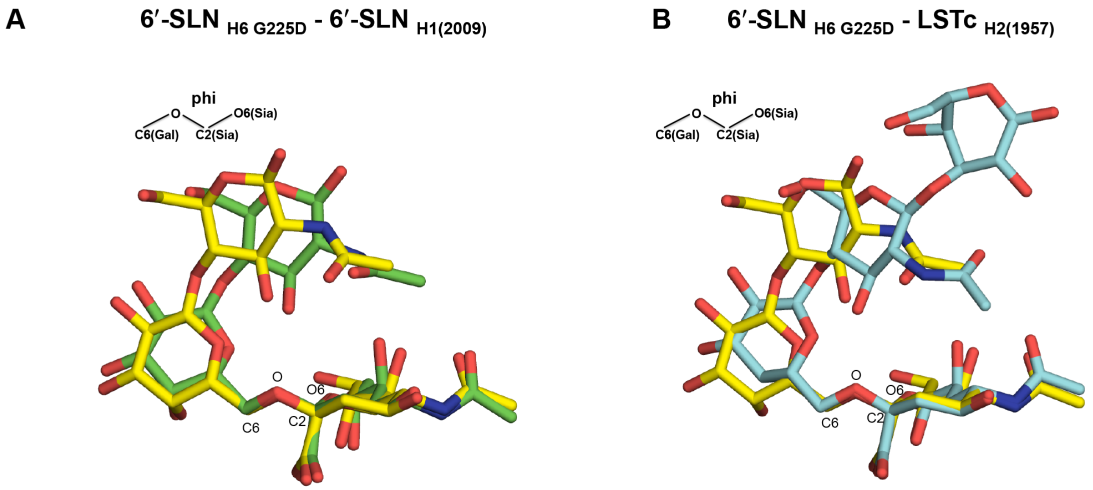

Figure S4. Structural comparison of human-type receptor conformations. Superposition of 6′-SLN receptor analog from the H6 G225D HA complex (yellow) compared to 6′-SLN human analogs from complex structures with HAs from (**A**) 2009 pandemic H1N1 (PDB 3UBN) and (**B**) 1957 pandemic H2N2 (PDB 2WR7). The superposition was done on Sia-1.

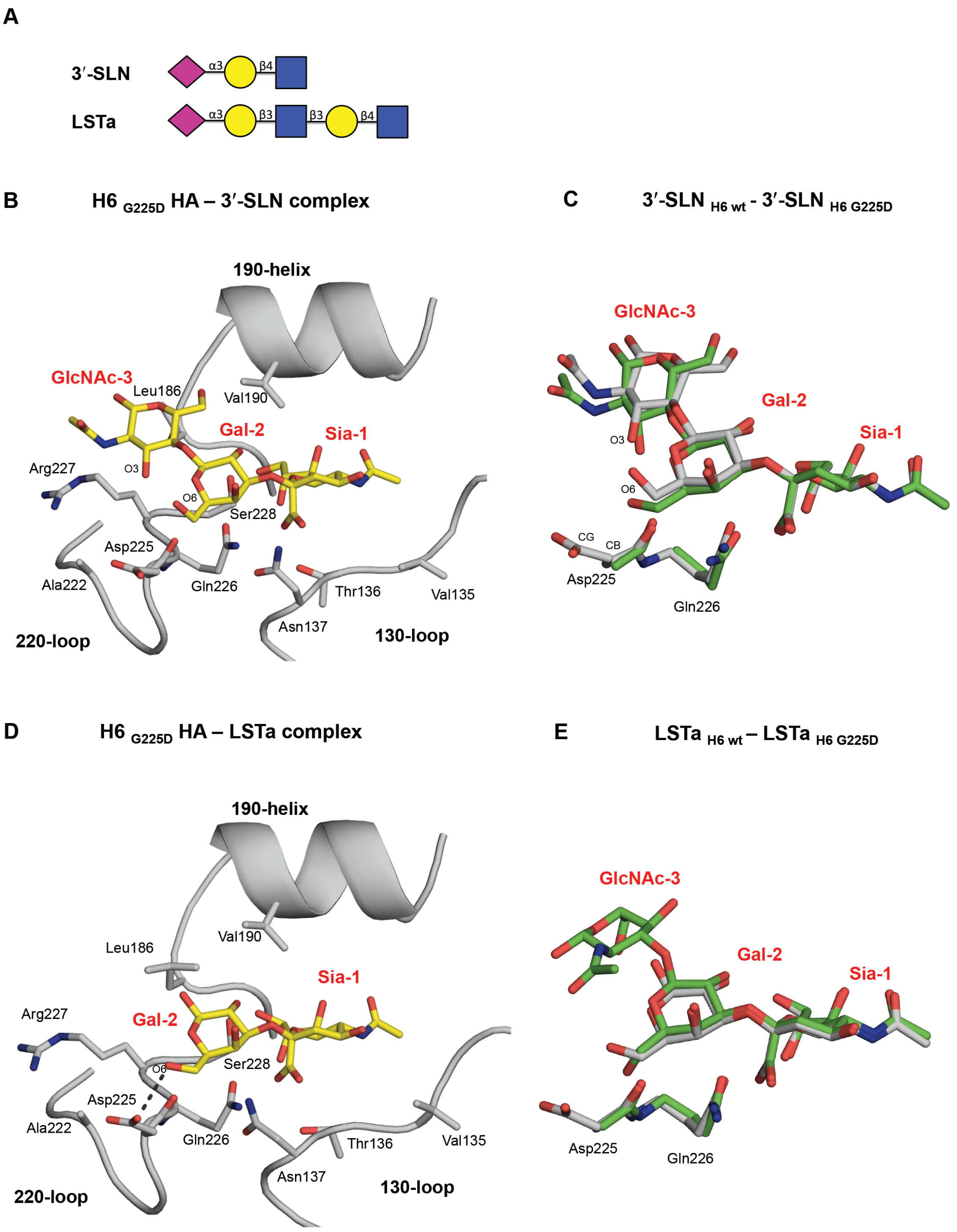

Figure S5. Crystal structures of the H6 G225D mutant in complex with avian receptor analogues. (**A**) The glycan structures of avian-type receptors 3′-SLN and LSTa. (**B** and **D**) Interactions between the H6 G225D RBS and the avian-type receptor analogues 3′-SLN (**B**) and LSTa (**D**). The receptor analogue is labeled, colored in yellow and shown in sticks. (**C** and **E**) Superposition of 3′-SLN (**C**) and LSTa (**E**) receptor analog from H6 G225D mutant complex (grey) compared to the H6 wild-type complex (green) indicates small conformational changes. The receptor analogues and the RBS Asp225 and Gln226 are labeled and shown in sticks.

REFERENCES

Davis IW, Leaver-Fay A, Chen VB, Block JN, Kapral GJ, Wang X, Murray LW, Arendall WB, 3rd, Snoeyink J, Richardson JS, Richardson DC (2007) MolProbity: all-atom contacts and structure validation for proteins and nucleic acids. *Nucleic Acids Res* **35:** W375-383.
